# Involvement of T Cell Immunity in Avian Coccidiosis

**DOI:** 10.3389/fimmu.2019.02732

**Published:** 2019-11-22

**Authors:** Woo H. Kim, Atul A. Chaudhari, Hyun S. Lillehoj

**Affiliations:** Animal Biosciences and Biotechnology Laboratory, U.S. Department of Agriculture, Beltsville Agricultural Research Center, ARS, Beltsville, MD, United States

**Keywords:** chicken, coccidiosis, T cells, avian immunology, host immunity

## Abstract

Avian coccidiosis is caused by *Eimeria*, which is an intracellular apicomplexan parasite that invades through the intestinal tract to cause devastating disease. Upon invasion through the intestinal epithelial cells, a strong inflammatory response is induced that results in complete villous destruction, diarrhea, hemorrhage, and in severe cases, death. Since the life cycle of *Eimeria* parasites is complex and comprises several intra- and extracellular developmental stages, the host immune responses are diverse and complex. Interferon-γ-mediated T helper (Th)1 response was originally considered to be the predominant immune response in avian coccidiosis. However, recent studies on other avian T cell lineages such as Th17 and T regulatory cells have implicated their significant involvement in maintaining gut homeostasis in normal and disease states including coccidiosis. Therefore, there is a need to understand better their role in coccidiosis. This review focuses on research findings concerning the host immune response induced by avian coccidiosis in the context of T cell immunity, including expression of T-cell-related cytokines and surface molecules that determine the phenotype of T lymphocytes.

## Introduction

Avian coccidiosis is caused by intracellular protozoan parasites that belong to several different species of *Eimeria* (1, 2). This apicomplexan parasite invades intestinal epithelial tissues and causes severe damage in birds, resulting in enormous economic losses in the poultry industry. The major challenge in coccidiosis control is the diversity among several *Eimeria* species that target different specific regions of the intestine.

The coccidia exhibit a complex life cycle comprising both intracellular and extracellular stages as well as asexual and sexual reproduction (3, 4). The life cycle mainly consists of an exogenous stage, characterized by excretion of unsporulated oocysts, and endogenous stage of schizogony (asexual reproduction) and gametogony (sexual differentiation) (5, 6). During the exogenous stage, the unsporulated oocysts become sporulated (with four sporocysts, each containing two sporozoites) under the influence of external environmental factors such as moisture, oxygen, and warmth. The endogenous stage occurs inside the host, which involves several stages of asexual reproduction followed by sexual reproduction, fertilization, and shedding of the unsporulated oocysts. In general, two to four generations of asexual reproduction are followed by the sexual phase, in which zygote formation takes place that eventually matures into oocysts that are released in the intestinal mucosa and finally shed into feces (7). The coccidia life cycle is usually short (4–6 days depending on several different species) and production of sporulating oocysts can easily increase the infectivity of the parasites in a large population of chickens. After ingesting the sporulated oocysts, excystation of oocysts occurs in the gizzard and the sporozoites are released, invade the intestinal cells, and cause severe damage as the reproductive cycle of the parasite begins. As a result, symptoms such as bloody diarrhea and reduced body weight and feed intake are observed in the birds.

Upon exposure to developing schizonts, anti-*Eimeria* immunity develops and is subsequently boosted by multiple re-exposures to oocysts (7). The immunity to avian coccidiosis can be categorized as innate and adaptive (8). As a first line of defense, the innate immune response is activated in response to the conserved antigens. Innate immune responses include recognition of conserved pathogen-associated molecular patterns (PAMPs) by pattern recognition receptors (PRRs) such as Toll-like receptors (TLRs) (5, 9, 10). A major TLR ligand, profilin, is expressed in all the developmental stages of the life cycle of several *Eimeria* parasites and is conserved (11). Such ligands induce a robust innate response such as immune cell proliferation and cytokine production. The cells involved in innate immune responses to *Eimeria* parasites at different phases are natural killer (NK) cells, dendritic cells, epithelial cells, heterophils, and macrophages. In particular, macrophage migration inhibitory factor plays a crucial role in mediating innate immunity in coccidiosis (12).

On the other hand, adaptive immunity is specific and regulates the antigen-specific immune responses to prevent colonization and growth of the pathogen inside the host. Like mammals, two major lymphocyte types, B cells (producing surface immunoglobulins) and T cells (T cell receptors), are the major components of adaptive immune responses in birds (13). Anticoccidial antibodies in serum and mucosal secretions have been reported in avian coccidiosis (13). Although B cell depletion studies (14) have revealed that antibodies do not play a specific role in anticoccidial protective immunity, other studies have emphasized the importance of passively transferred humoral immunity in *Eimeria* infection in chickens (15–18). Cell-mediated immunity in avian coccidiosis is characterized by antigen-specific or non-specific activation of several immune cells such as T cells, NK cells, and macrophages. The CD4^+^ T helper (Th) cells and CD8^+^ cytotoxic T lymphocytes (CTLs) are the two major T-cell subsets that are involved in anticoccidial immunity (19–22). Although the role of several T-cell subpopulations in avian coccidiosis remains to be elucidated, T cells are the most important for protection against *Eimeria* infections in birds.

In this article, we reviewed the historical progress of immunological studies on the host immune response to avian coccidiosis, with an emphasis on recent findings in the understanding of the complexity of T-cell immune responses in avian coccidiosis, especially those mediated by Th17 and T regulatory (Treg) cells.

## Development Of Immunology In Avian Coccidiosis

Since the first report of chicken coccidiosis in the cecum in the late eighteenth century (1), immunity to several *Eimeria* parasites has been investigated thoroughly. An important contribution from Rose and colleagues (23) defined the basic principles of avian immunity to coccidial parasites in terms of specificity, wherein one species of *Eimeria* offers little protection against heterologous challenge with other species. Over the past few decades, studies focusing on investigating the role of avian immunity in response to various coccidial parasites has shown promising developments toward better understanding of avian immunity to coccidiosis (20, 24, 25). From all these studies, it was apparent that out of the two types of immunity, cellular immunity was more important than humoral immunity in coccidiosis, as the later offered little protection against the infection. Early investigations in both mammalian and avian species have revealed that the cellular immune responses through T cells and their associated cytokines play an important role in anticoccidial immunity (2, 26, 27). Acquired immunity to murine coccidiosis is attributed more to T cells than B cells (26). Several immune cell types including NK cells, dendritic cells, and macrophages are involved in innate immune responses to avian coccidiosis (8, 27). B-cell-deficient chickens have shown increased oocyst production after primary infection with *Eimeria* species. However, secondary infection does not yield clinical coccidiosis in the bursectomized chickens due to the protective immunity acquired by the primary infection (8). This indicates that the anticoccidial immunity acquired after primary infection is B cell independent. It is also apparent that chicken coccidiosis can be prevented by adaptive transfer of peripheral blood lymphocytes and splenocytes from *Eimeria-*infected chickens in the syngeneic recipients (28). Subsequently, the T-cell immunosuppressant cyclosporin A abolished the protective immunity offered by *Eimeria* re-infection, thus further emphasizing the integral role of cellular immune mechanisms in chicken coccidiosis (14).

The early findings indicated that T cells serve as a key factor to mediate anticoccidial immunity in chickens (8, 14). Greater numbers of CTLs expressing CD8 cell surface antigen were predominantly observed in chickens after primary infection (29–31). Furthermore, the differential role of CD4^+^ and CD8^+^ T lymphocytes in offering resistance to primary and secondary coccidial infection was also reported (32, 33). Increased populations of T cells are linked to elevated production of proinflammatory cytokine interferon (IFN)-γ, which has an immunoregulatory effect (34), as well as inhibiting intracellular development of the parasite (35, 36). The role of T cells in mediating host immunity to coccidiosis became more evident when flow cytometric analysis of intestinal epithelial lymphocytes (IELs), using lymphocyte-specific immune reagents, revealed their significance in innate immunity in naïve chickens and adaptive immunity in previously infected chickens (19, 37, 38). More studies showed that different IEL subtypes are involved in anti-*Eimeria* defense in the gut (39, 40). Research over the past several years has shown that, as a part of protective immunity against avian coccidiosis, T cells produce numerous secretions besides IFN-γ, such as cytokines interleukin (IL)-1, IL-2, IL-4–6, IL-8, IL-10, IL-12, IL-13, and IL-15–18, tumor necrosis factor (TNF)-α, lipopolysaccharide-induced TNF-α factor (LITAF), TNF-α superfamily 15 (TNFSF15), transforming growth factor (TGF)-β1–4, and granulocyte–macrophage colony-stimulatory factor (GM-CSF). All these findings are based on research oriented toward investigating the immunoregulatory responses of these molecules after primary and/or secondary coccidiosis (40–60). More recent work has indicated the involvement of TLR4 and TLR15 as a part of the innate immune response to *Eimeria* infection (61). IL-17 also contributes to host immunopathology in response to experimental infection (62). The immunoproteomics analysis of three *Eimeria* species has identified several immunodominant antigens from these three species that could provide a useful breakthrough in exploring anticoccidial immunity, as some of these molecules cause profuse inflammatory and cellular immune response that contribute to pathogenesis and severity of infection (3, 63). Additionally, research on anticoccidial vaccines and natural alternatives has explored the immunobiology of coccidiosis in poultry (7). All these findings show the immunoregulatory effect of vaccines or several naturally occurring anti-inflammatory products such as curcumin and *Allium hookeri*. These findings also provide a useful insight into immunoregulation in avian coccidiosis, however, this is outside the scope of this review and has been reviewed previously (7). Much of this work has focused on immunomodulation by dietary ingredients in experimental *Eimeria* infections (64, 65).

Besides the above information, immunological variation among the different strains of the same *Eimeria* species has also been reported in chickens (66, 67). These findings show the characteristic intraspecific variations attributed to the biological features of *Eimeria*, such as morphology of oocysts, pathogenicity, and sensitivity to drugs (68). This inter- and intraspecies variation has been recently defined with the help of more advanced molecular approaches such as random amplification of polymorphic DNA–PCR, and restricted or amplified fragment length polymorphism (69). Analysis of these variations has led to the identification of several strain-specific immunoprotective antigens (70, 71). Similarly, more recent findings have also highlighted the variation in immune responses to *Eimeria tenella* infection in genetically distinct chicken lineages (72).

## Role Of IFN-γ-Mediated Immunity In Avian Coccidiosis

Among all the cytokines mentioned above, IFN-γ is a major cytokine that has anticoccidial effects (73). In mammals, parasitic infections are often characterized by increased levels of IFN-γ. Similarly, the functional role of this cytokine in *Eimeria* infections has been studied thoroughly (34, 74, 75). Until the cDNA cloning of chicken IFNs revealed the independent existence of type I (76) (IFN-α) and type II (77) (IFN-γ) IFNs, most of the findings on the role of IFN-like activity in *Eimeria* infections were believed to be associated with IFN-γ. All these IFN-dependent activities inhibit the invasion or development of *Eimeria* in cultured cells *in vitro* (73, 78). *In vivo* studies have also revealed the anticoccidial IFN-like activity in *Eimeria*-infected birds (79, 80). The specific involvement of IFN-γ in anti-*Eimeria* immunity was later described by Breed et al. who showed that IFN-γ was produced specifically after stimulation of peripheral blood lymphocytes from *Eimeria-*infected birds (31). It was then discovered that mitogen- or antigen-stimulated specific T cells circulating in the blood of *Eimeria*-infected chickens specifically produced IFN-γ (81). Based on these findings, it was proposed that T-cell priming might occur at the site of infection, resulting in production of IFN-γ at the infection site, thus regulating anticoccidial immunity (81). It was also hypothesized that CD8^+^ cells produce IFN-γ, which is involved in immunoregulation in primary coccidiosis (81). These findings were extended by Rothwell et al. who showed that IFN-γ-producing cells were present in blood and the spleen and may migrate from the spleen after secondary infection (82). *In situ* hybridization has shown that, following *Eimeria* challenge, IFN-γ is produced by the cells (predominantly T cells) at the site of infection (cecum) and by splenocytes (82). Several studies have shown the potential application of IFN-γ in protecting against *Eimeria* infections (36, 83, 84). Birds immunized with recombinant IFN-γ show increased body weight gain during infection with *Eimeria acevirulina* (36, 83). Also, the development of *E. tenella* is inhibited by IFN-γ *in vitro* (36, 84). When chicken cells are treated with recombinant IFN-γ, intracellular development of *E. tenella* is inhibited, with no significant effect on sporozoite invasion of the cells (36). Similarly, *in vivo* administration of recombinant IFN-γ protects against *E. acevirulina* characterized by reduced oocyst production and increased body weight gain (36, 83).

Besides its immunoregulatory or immunoprotective effect against chicken coccidiosis, IFN-γ has also been shown to have an adjuvant effect on coccidial vaccine in *Eimeria-*infected chickens (85). The adjuvant effect of IFN-γ is characterized by enhanced immune response to the vaccine antigen that induces a microbicidal effect to resolve the parasitic infection, thus increasing vaccine efficacy (85). Some DNA vaccines administered with IFN-γ increased the immunity at intestinal level and protected against avian coccidiosis (36, 86, 87). Recent studies have also indicated the beneficial effect of IFN-γ on anticoccidial DNA vaccine (88, 89). A chimeric vaccine constructed by fusion of genes encoding the *E. tenella* surface antigen, and IFN-γ alleviated the cecal lesions and improved the anticoccidial index in experimentally infected chickens, further suggesting the adjuvant effect of IFN-γ (89). Thus, all these efforts indicate the anticoccidial role of IFN-γ, direct, or as an adjuvant, and underline the significance of IFN-γ in anticoccidial immunity.

## Th17 Cells And Their Cytokines In Avian Coccidiosis

Besides the response elicited by IFN-γ-mediated Th1 cells against avian coccidiosis, the other CD4 T-cell subsets have also been studied since the discovery of their homologs in mammals (42). A lineage of IL-17-producing CD4^+^ T helper (Th)17 cells that are distinct from the previously well-characterized Th1/Th2 paradigm, has emerged and is involved in proinflammatory responses in various autoimmune diseases and infections (90). The biological activities of IL-17 as a signature cytokine of Th17 cells include recruitment of neutrophils, stimulation of antimicrobial peptide production, such as β-defensins and mucins, as well as induction of cytokines and chemokines, in particular IL-6, CXCL8 and GM-CSF (91). Chicken IL-17 isolated from *Eimeria*-infected IELs exerts a proinflammatory role in coccidiosis (8). The exact role of Th17 cells in chicken is poorly understood due to the lack of immunological reagents. This section describes studies that focused on the role of IL-17 as a signature cytokine in Th17 cells in chicken coccidiosis. Following infection by *E. acervulina* or *E. maxima*, IL-17 mRNA levels were increased in IELs compared to uninfected controls (40). In *E. tenella* infection, IL-17 expression in IELs was downregulated, except in the latter stage of infection (39). Similarly, Kim et al. reported that chicken IL-17 expression was downregulated in inflamed intestinal tissue following *E. tenella* infection, and treatment with IL-17 or IL-17F induced expression of proinflammatory cytokines in chicken fibroblasts (92). These results suggest that chicken coccidiosis induces IL-17 expression in the gut and is dependent on the species of *Eimeria*. Th17 response can play both protective and pathological roles in protozoan infections. The cloning of IL-17 receptor A (IL-17RA), which binds IL-17A and IL-17F in chickens, has revealed that *Eimeria* infection downregulates expression of IL-17RA, and modulation of this receptor facilitates the host to reduce intestinal pathogenesis amplified by IL-17/IL-17RA signaling. Several authors have proposed that Th17 cells or IL-17 promote pathogenesis in leishmaniasis, toxoplasmosis, and *Eimeria falciformis* infection (93–95), whereas others have demonstrated that they are involved in protective immunity against trypanosomiasis, toxoplasmosis and *Pneumocystis carinii* infection (96, 97). Recent evidence seems to support a role for Th17 cytokines in host immunopathology in coccidiosis in chickens. Treatment with IL-17 neutralizing antibody in *E. tenella* infection induces lower heterophil recruitment, inflammatory cytokine expression, and parasite burden in the intestinal tract, resulting in enhanced body weight gain, reduced oocyst production in feces, and intestinal lesions (62). IL-17 is also involved in the initiation and migratory response of epithelial cells during intracellular development, and maturation of parasites, contributing to pathogenesis in the intestinal tract. Following *E. tenella* infection, chickens treated with IL-17 neutralizing antibody have a reduced number of second-generation schizonts and cecal lesions (98).

## Anti-inflammatory IL-10 And Treg Cells In Avian Coccidiosis

Treg cells are a subset of T cells involved in immunosuppression. Mammalian Treg cells have the phenotype CD4^+^CD25^+^FoxP3^+^ (99). In chickens, the ortholog of mammalian FoxP3 has yet to be identified, although there is a report of an avian *foxp3* gene (100, 101). Thus, CD4^+^CD25^+^ T cells in chickens have been characterized as Treg cells showing suppression of activated immune cells (102). These cells produce high amounts of IL-10, TGF-β, CTLA-4, and LAG-3, as in mammals (103). IL-10 showed 29-fold higher expression in CD4^+^CD25^+^ cells compared to CD4^+^CD25^−^ cells and its immunosuppression in chickens has been extensively studied (102). In coccidiosis, IL-10 is considered to play an important role in evasion of the host immune response. One possible mechanism to explain its role in coccidiosis is that coccidial parasites have evolved to stimulate Treg cells to express IL-10, and it helps parasites to facilitate invasion and survival in chickens through suppression of the IFN-γ-related Th1 response that is critical for protective immunity against coccidial parasites. Two inbred lines of chickens that differ in their resistance or susceptibility to *Eimeria* infection have revealed that expression of IL-10 is the major difference between the two lines. Expression of IL-10 is highly induced in susceptible chickens among the genes related to different Th lineages, such as IFN-γ for Th1, IL-4 for Th2, and IL-10 and TGF-β for Treg cells, while IL-10 is suppressed in the age-matched resistant line (46). *Eimeria*-infected chickens treated with IL-10 neutralizing antibody show improved growth rate compared to those with control antibody but it has no effect on fecal oocyst production (104, 105). Morris et al. reported that supplementation of vitamin D induced IL-10 expression as well as Treg cells and showed decreased production losses associated with coccidial infection (106). These results indicate that regulation of the protective immune response to *Eimeria* infection by Treg cells is critical, and IL-10 plays a role in pathogenesis in chicken coccidiosis. We recently identified that Treg cells could help to reduce pathology in *Eimeria*-infected intestine by suppression of Th17 cells that induce tissue inflammation. Increased expression of CD4^+^CD25^+^ Treg cells has been found in *E. tenella*-infected chickens with increased IL-10 expression. After treatment with aryl hydrocarbon receptor such as 3,3′-diindolylmethane, Treg cells are increased in the intestine, whereas CD4^+^IL-17^+^ Th17 cells are suppressed. We have also found that generation of Th17 cells is suppressed by Treg cells, which leads to reduced pathogenicity in chicken coccidiosis (107).

## Concluding Remarks

It is the consensus that the Th1 response is the most efficient host response in avian coccidiosis. However, studies on other aspects like Th17 and Treg responses are also important because the immune responses are not independent, but rather they are connected and work together in an integrated immune system. It is becoming clear that the outcome of an inflammatory process caused by infection depends on the balance of responses by several components of the immune system of particular relevance is the interplay between Treg and Th17 cells during immunoinflammatory events (108). Compared to mammalian immunology, little is known about the role of T cells in chickens, although the number of reports on coccidiosis is steadily growing. To understand better immunity against chicken coccidiosis, it is necessary to know how T cells are modulated and how they interplay since this intracellular pathogen predominantly induces a T-cell-associated immune response that involves several types of T cells. In regard to controlling coccidiosis, the best way might be development of alternatives to antibiotics because most effective anticoccidial drugs that produce resistance or residues will be banned from the market in the future. Understanding the mechanism of how chickens respond to *Eimeria* will lead to new approaches to control coccidiosis.

## Author Contributions

All authors listed have made a substantial, direct and intellectual contribution to the work, and approved it for publication.

### Conflict of Interest

The authors declare that the research was conducted in the absence of any commercial or financial relationships that could be construed as a potential conflict of interest.
